# Distinctive HBV Replication Capacity and Susceptibility to Tenofovir Induced by a Polymerase Point Mutation in Hepatoma Cell Lines and Primary Human Hepatocytes

**DOI:** 10.3390/ijms22041606

**Published:** 2021-02-05

**Authors:** Ah Ram Lee, Ju-Yeon Cho, Jong Chul Kim, Mehrangiz Dezhbord, Soo Yeun Choo, Chang Hyun Ahn, Na Yeon Kim, Jae Jin Shin, Soree Park, Eun-Sook Park, Juhee Won, Dong-Sik Kim, Jeong-Hoon Lee, Kyun-Hwan Kim

**Affiliations:** 1 Department of Precision Medicine, School of Medicine, Sungkyunkwan University, Suwon 16419, Korea; ahram2g@naver.com (A.R.L.); rlawhdcjf95@naver.com (J.C.K.); m.dezhbord@yonsei.ac.kr (M.D.); michellechoo@naver.com (S.Y.C.); quek689@gmail.com (C.H.A.); michaela3310@naver.com (N.Y.K.); jaejin362@kakao.com (J.J.S.); rhd37@naver.com (S.P.); 1wonjuhee@hanmail.net (J.W.); 2 Department of Internal Medicine, College of Medicine, Chosun University, Gwangju 61452, Korea; 3 Department of Pharmacology, School of Medicine, Konkuk University, Seoul 05029, Korea; espark97@gmail.com; 4 Department of Surgery, Division of HBP Surgery and Liver Transplantation, College of Medicine, Korea University, Seoul 02841, Korea; kimds1@korea.ac.kr; 5 Department of Internal Medicine and Liver Research Institute, College of Medicine, Seoul National University, Seoul 03080, Korea; pindra@empas.com

**Keywords:** hepatitis B virus, tenofovir disoproxil fumarate (TDF), reverse transcription, nucleos(t)ide analog, drug resistance

## Abstract

Tenofovir disoproxil fumarate (TDF) has been regarded as the most potent drug for treating patients with chronic hepatitis B (CHB). However recently, viral mutations associated with tenofovir have been reported. Here, we found a CHB patient with suboptimal response after more than 4 years of TDF treatment. Clonal analysis of hepatitis B virus (HBV) isolated from sequential sera of this patient identified the seven previously reported TDF-resistant mutations (CYELMVI). Interestingly, a threonine to alanine mutation at the 301 amino acid position of the reverse-transcriptase (RT) domain, (rtT301A), was commonly accompanied with CYELMVI at a high rate (72.7%). Since the rtT301A mutation has not been reported yet, we investigated the role of this naturally occurring mutation on the viral replication and susceptibility to tenofovir in various liver cells (hepatoma cells as well as primary human hepatocytes). A cell-based phenotypic assay revealed that the rtT301A mutation dramatically impaired the replication ability with meaningful reduction in sensitivity to tenofovir in hepatoma cell lines. However, attenuated viral replication by the rtT301A mutation was significantly restored in primary human hepatocytes (PHHs). Our findings suggest that the replication capability and drug sensitivity of HBV is different between hepatoma cell lines and PHHs. Therefore, our study emphasizes that validation studies should be performed not only in the liver cancer cell lines but also in the PHHs to understand the exact viral fitness under antiviral pressure in patients.

## 1. Introduction

Despite the availability of an effective vaccine and potent antiviral drugs, chronic hepatitis B virus (HBV) infection remains a major health concern worldwide, with an estimated 350 million infected individuals and 786,000 deaths each year [1,2].

Several nucleos(t)ide analogs (NAs) [3] including lamivudine, adefovir (ADV), telbivudine, entecavir (ETV), tenofovir disoproxil fumarate (TDF), and tenofovir alafenamide (TAF) have been approved to treat chronic hepatitis B infection [3,4]. These antiviral drugs target the reverse transcriptase (RT) domain of HBV polymerase and interfere with DNA synthesis [5]. Despite the probability of emerging drug resistance against NAs in long-term treatment, ETV, TDF, and TAF are nonetheless reported to be highly potent with low rates of resistance development (1–2%, 0%, and 0% after 5 years of treatment, respectively) [6,7]. Accordingly, these drugs have been recommended as the first-line treatment by recent practice guidelines [3,4,8,9].

Tenofovir is the active metabolite of prodrugs, TDF and TAF. TAF has the same efficacy as TDF but has been developed to reach a high intracellular concentration (>90%) with improved renal and bone safety compared to TDF [10]. Despite its high genetic barrier to resistance, we have previously reported that a novel quadruple mutation (CYEI; rtS106C (C), rtH126Y (Y), rtD134E (E), and rtL269I (I)) is associated with tenofovir resistance [11]. Although the HBV mutants harboring CYEI mutations were dominantly found in two TDF-resistant patients, a rtT301A (A) mutant together with CYEI mutations (CYEIA) was also identified from one patient as a minor portion [11]. While continuing the study of tenofovir resistance, this mutation was found dominantly in the serum of a chronic hepatitis B (CHB) patient without complete virological suppression after more than 4 years of TDF treatment, which led to the further progress of this study.

Therefore, in this study, we aimed to identify the role of a naturally occurring rtT301A mutation in HBV replication ability and susceptibility to tenofovir, using patient-derived HBV RT mutants and artificially constructed clones. Moreover, we tested the effect of the rtT301A mutation on replication capability and drug sensitivity in hepatoma cell lines and primary human hepatocytes (PHHs). We found that the replication capability and drug sensitivity are different between hepatoma cell lines and PHHs. Our study suggests that the validation studies must be performed not only in liver cancer cell lines but also in PHHs to understand the exact replication and drug resistance characteristics of HBV mutants in patients.

## 2. Results

### 2.1. Mutation Profile of HBV RT Domain Cloned from a TDF-Treated Patient

**Figure 1:**
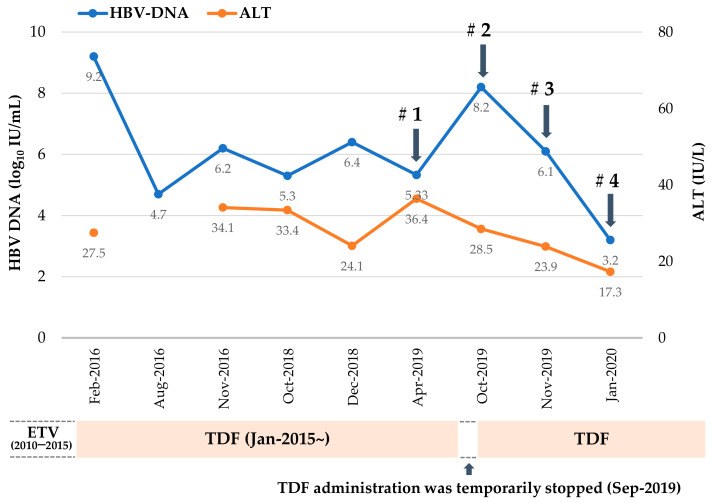
Clinical course of a chronic hepatitis B patient with incomplete virological response after 9 years of antiviral treatment. Blood samples (#1–#4) were collected at indicated time points and hepatitis B virus (HBV) DNA (IU/mL) and ALT (IU/mL) levels were measured. At Aug-2016, only DNA level was measured. TDF, tenofovir disoproxil fumarate; ALT, alanine aminotransferase.

Clinical course of the CHB patient with incomplete virological response after 9 years of sequential antiviral treatment with ETV and TDF is profiled in Figure 1. As indicated by arrows, blood samples were obtained four times during TDF treatment, and HBV DNA was isolated from patient serum to analyze mutations in the RT domain of HBV polymerase gene. The mutant HBV 1.2mer replicons were constructed using patient-derived RT sequence and compared to that of wild type (WT) (Table 1).

In the first sample, isolated in Apr 2019, (Serum #1), seven common CYELMVI mutations that were previously reported as tenofovir-resistant mutations in our preceding study [11] were identified. Unexpectedly, CYELMVI was predominantly selected in the absence of viral breakthrough and at the nadir point of the viral load during the treatment course. In September of 2019, the patient stopped TDF medication by her own decision for 1 month whilst being treated for hydrocephalus. The viral load checked in October 2019 (Serum #2) confirmed virological breakthrough with increases up to more than 8 log10 IU/mL. The dominant population was WT HBV, and no drug-resistant mutations were observed. HBV DNA decreased consistently thereafter, but the CYELMVI mutant only began to appear three months later in January 2020 (Serum #4). Interestingly, clonal analysis from all four sample collections revealed that 72.7% (8 out of 11 clones) of CYELMVI mutants harbored a novel rtT301A (A) substitution (Table 1).

Therefore, since rtT301A mutation was frequently accompanied by tenofovir-resistant mutations (CYELMVI) while distinct viral breakthrough did not occur, we sought to identify the effect of this particular substitution in RT sequence on HBV replication ability and drug susceptibility.

### 2.2. Patient-Derived HBV Mutant Harboring CYELMVI Mutation Is Resistant to Tenofovir Treatment

**Figure 2:**
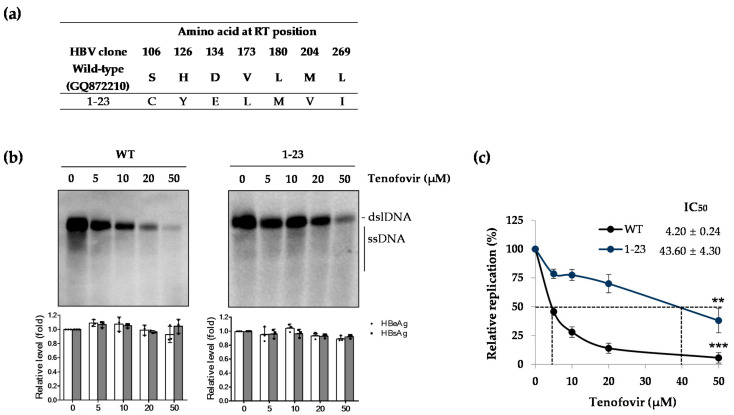
Replication competence and in vitro drug susceptibility of a representative patient-derived RT mutant. (**a**) Sequence of a representative RT mutant clone obtained from #1 serum of a TDF-treated patient. (**b**) In vitro tenofovir susceptibility assay of WT and Clone 1-23. The constructed HBV 1.2mer replicons were transfected into Huh7 cells, and tenofovir was administered every day in a dose-dependent manner as indicated in μM. Four days later, the levels of HBV DNA and secreted HBeAg/HBsAg were analyzed by Southern blot and enzyme-linked immunosorbent assay (ELISA), respectively. Secreted antigen levels were assessed to confirm the transfection yield. (**c**) IC_50_ values determined by Southern blot using HBV-specific digoxigenin (DIG) labeled probe in a Quantimager. ** *p* < 0.01; *** *p* < 0.001. Data in (**b**,**c**) were obtained from three independent experiments (mean ± SD). RT, reverse transcriptase; WT, wild-type; HBeAg, HBV e antigen; HBsAg, HBV s antigen.

We constructed the HBV 1.2mers where the WT RT gene was replaced with the patient-derived RT region and, therefore, obtained 22 clones from Serum #1 (from Clones 1-1 to 1-38 in Table 1). To evaluate the drug susceptibility of RT mutants obtained from Serum #1, Clone 1-23 was selected as the most representative clone carrying CYELMVI tenofovir-resistant mutations (Figure 2a). This clone was transiently transfected into the Huh7 cell, and the level of drug susceptibility as well as secreted HBV antigens were examined. As shown in Figure 2b, the WT HBV DNA level was reduced in a dose-dependent manner with tenofovir treatment (left panel), whereas Clone 1-23 was resistant to tenofovir. Thus, the replication level was roughly maintained up to the maximum drug dose, 50 μM (right panel), which was consistent with our previous report [11]. There was no significant difference in the secreted HBV e antigen (HBeAg) and HBV s antigen (HBsAg) levels, which implies that the reduced HBV DNA was not affected by the transfection yield of each clone and tenofovir has no role in diminishing viral transcription and translation. IC_50_ values, calculated based on Southern blot, for WT and Clone 1-23 were 4.20 ± 0.24 and 43.60 ± 4.3 μM, respectively (Figure 2c). Substantial (more than 10.3-fold) differences in IC_50_ values of the WT and mutant confirmed that CYELMVI mutations significantly contributed to the development of tenofovir resistance.

### 2.3. Effect of Patient-Derived HBV RT Mutants Harboring the rtT301A Mutation on Replication Capacity and Tenofovir Resistance in Hepatoma Cell Lines

**Figure 3:**
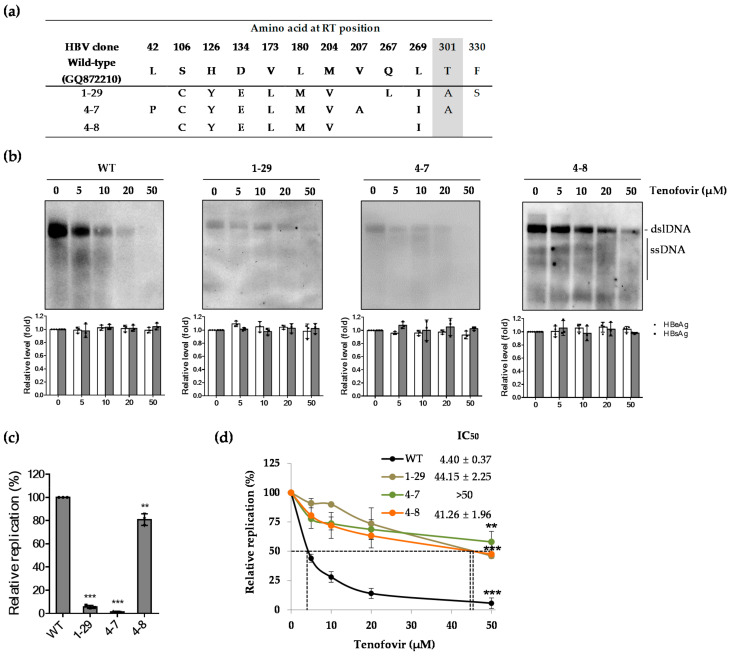
Effect of the naturally occurring rtT301A mutation on replication ability and tenofovir resistance in Huh7 cells. (**a**) Representative tenofovir-resistant clones (with or without the rtT301A mutation) obtained from the TDF-treated patient. (**b**) In vitro tenofovir susceptibility assay by Southern blot in Huh7 cells. The levels of secreted HBeAg and HBsAg were analyzed by ELISA. (**c**) The replication capacity of each clone was determined by quantitative real-time PCR, compared to that of WT. ** *p* < 0.01; *** *p* < 0.001. (**d**) IC_50_ values were measured by quantitative real-time PCR. The level of HBV replication without drug treatment was set to 100%. ** *p* < 0.01; *** *p* < 0.001. Data in (**b**–**d**) were obtained from three independent experiments (mean ± SD).

The role of the novel rtT301A mutation was investigated using three representative clones (Clones 1-29, 4-7, and 4-8) carrying tenofovir-resistant mutations (CYELMVI), of which Clones 1-29 and 4-7 carried an additional rtT301A mutation (Figure 3a). Southern blot analysis was performed to assess the drug susceptibility of the three clones. Clone 4-8 showed resistance to tenofovir as compared to the WT. Southern blot of Clones 1-29 and 4-7 demonstrated low levels of HBV replication, indicating that the substitution mutants resulted in a major loss of replication competence (Figure 3b). However, the levels of HBsAg and HBeAg in culture supernatants of transfected Huh7 cells were not altered by tenofovir treatment, indicating that regardless of low replication capacity, production of viral RNAs and antigens of each clone were not affected by the rtT301A substitution. The relative replication level of each clone determined by quantitative real-time polymerase chain reaction (PCR) was 5.50% ± 1.5% and 2.50% ± 0.5% for Clones 1-29 and 4-7, respectively, compared to WT HBV (Figure 3c). In order to overcome the difficulty of quantifying replicative DNA due to low replication level, IC_50_ values were analyzed by quantitative real-time PCR, which is a more sensitive method for detection of viral DNA at low levels. As a result, IC_50_ values of patient-derived HBV RT mutants harboring tenofovir-resistant mutations were 44.15 ± 2.25, >50, and 41.26 ± 1.96 μM for Clones 1-29, 4-7, and 4-8, respectively. In particular, Clones 1-29 and 4-7 showed slightly increased resistance to tenofovir (Figure 3d), suggesting that the rtT301A substitution may diminish replication capability and increase tenofovir resistance simultaneously.

**Figure 4:**
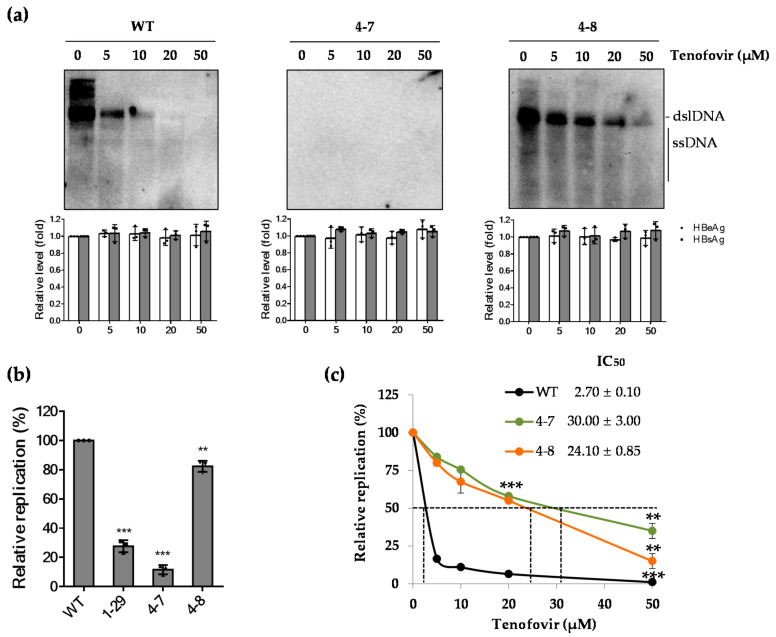
Effect of the naturally occurring rtT301A mutation on replication ability and tenofovir resistance in HepG2 cells. (**a**) In vitro tenofovir susceptibility of indicated clones was determined by Southern blot in HepG2 cells. The levels of secreted HBeAg and HBsAg were analyzed by ELISA to confirm transfection yield. (**b**) The replication capacity was evaluated by quantitative real-time PCR, compared to that of WT which was set to 100%. ** *p* < 0.01; *** *p* < 0.001. (**c**) IC_50_ values were determined by quantitative real-time PCR. ** *p* < 0.01; *** *p* < 0.001. All data were obtained from three independent experiments (mean ± SD).

To rule out the possibility that the effect of rtT301A mutation shown in Huh7 cell is not cell-type specific, we performed similar experiments in HepG2 cells. As demonstrated in Figure 4a, all clones secreted similar levels of antigens. However, the patient-derived clones (Clones 4-7 and 4-8) exhibited resistance to tenofovir treatment, and the replicative capacity of Clone 4-7 was dramatically lower than that of WT or Clone 4-8 in HepG2 cells, which was similarly observed in Huh7 cells (Figure 4b). In addition, IC_50_ values of WT, Clone 4-7, and Clone 4-8, determined by quantitative real-time PCR, were 2.7 ± 0.1, 30.0 ± 3.0, and 24.10 ± 0.85 μM, respectively (Figure 4c). In line with Huh7 data, Clone 4-7 showed higher resistance to tenofovir treatment. As similar results were obtained from both Huh7 and HepG2, the effect of rtT301A mutation maybe comparable among hepatoma cell lines, implying that that the naturally occurring novel rtT301A mutation impaired the replication competence and increased resistance to tenofovir.

### 2.4. The rtT301A Mutation Decreases Replication Capacity and Increases Tenofovir Resistance in Hepatoma Cell Lines

**Figure 5:**
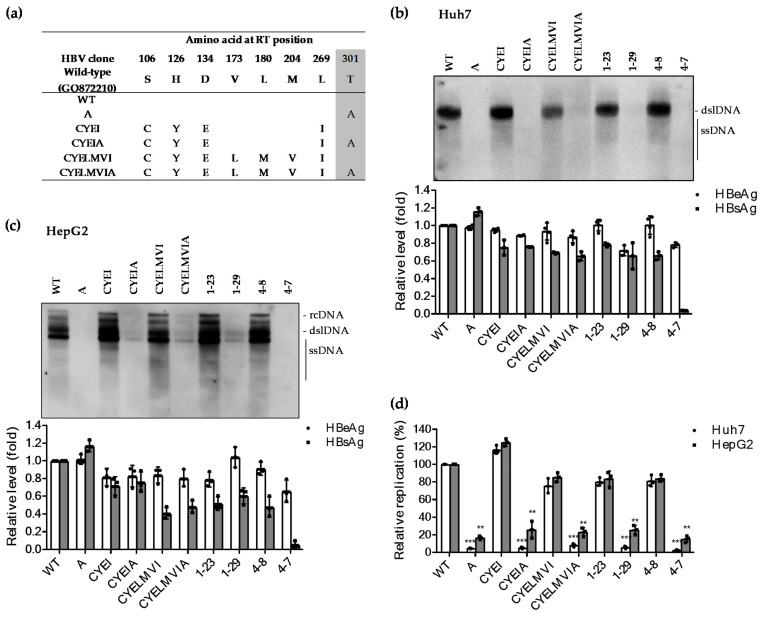

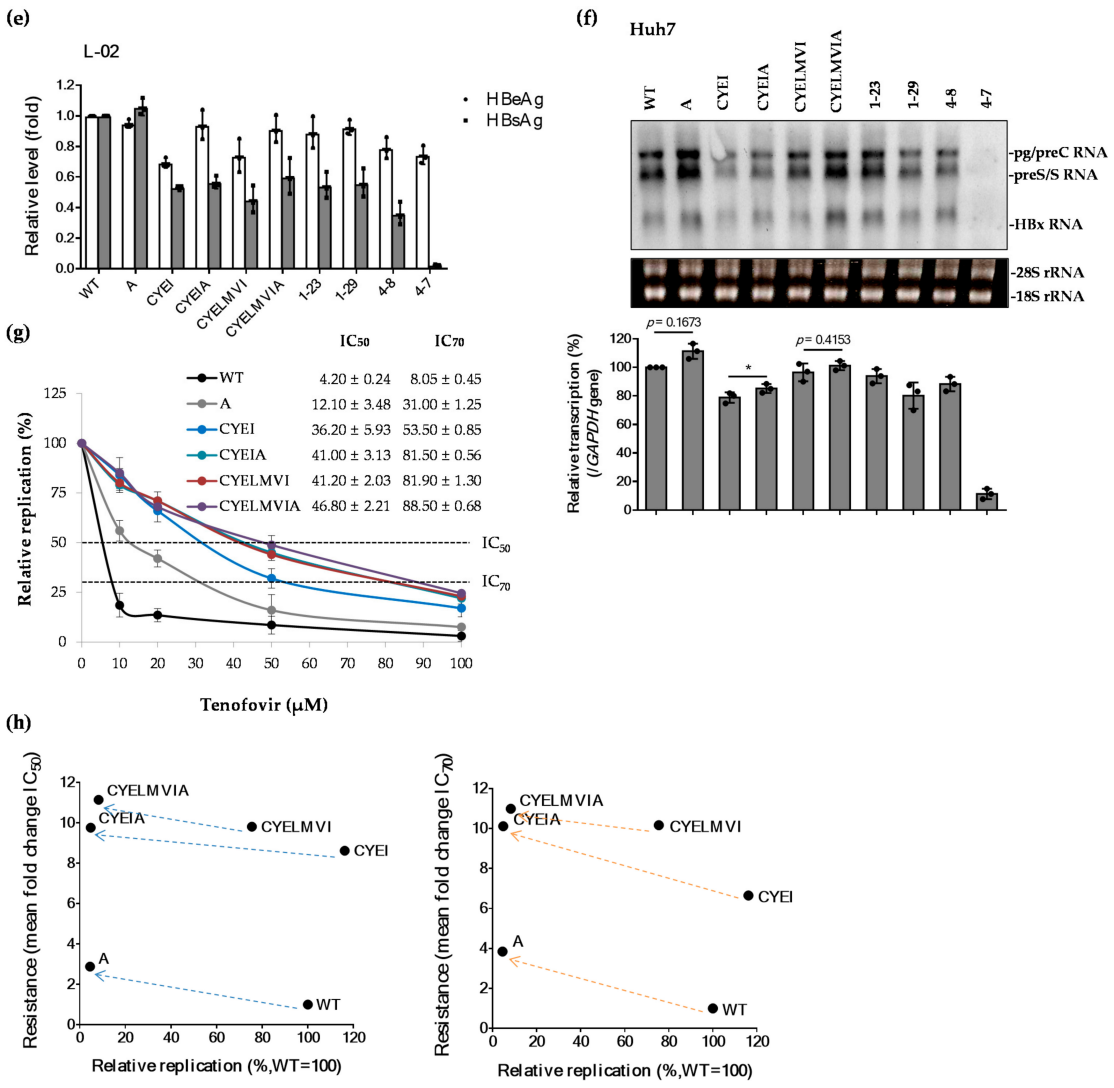
Effect of the rtT301A mutation on replication ability, viral transcription, and tenofovir resistance in different hepatoma cell lines. (**a**) A schematic representation of artificial mutant clones. The rtT301A mutation was introduced to each WT, CYEI, and CYELMVI clone a by site-directed mutagenesis. (**b**,**c**) The artificial and patient-derived HBV RT mutant clones were transfected into Huh7 (**b**) or HepG2 cells (**d**), and HBV replication was analyzed by Southern blot. The levels of secreted HBeAg and HBsAg were measured by ELISA. (**d**) The replication capacity was determined and compared in Huh7 and HepG2 cells by quantitative real-time PCR. The WT replication value was set to 100%. ** *p* < 0.01; *** *p* < 0.001. (**e**) The secreted HBeAg and HBsAg levels of each clone in L-02 cells. (**f**) HBV RNA levels of each clone was determined by Northern blot (upper) and quantified by quantitative real-time PCR (lower) in Huh7 cells. * *p* < 0.05. (**g**) Effect of the rtT301A mutation on susceptibility to tenofovir treatment. The IC_50_ and IC_70_ values of each clone were determined by quantitative real-time PCR in Huh7 cells. (**h**) Plots of IC_50_ (left) or IC_70_ (right) values vs. relative viral replication compared to the WT. Arrows indicate the decreased replication capacity and increased tenofovir resistance by rtT301A mutation. Data in (**b**–**h**) were obtained from three independent experiments (mean ± SD). pg/preC RNA, pregenomic/precore RNA.

To confirm that the rtT301A mutation is associated with viral replication and tenofovir resistance, we introduced the rtT301A mutation into three replicons: WT, CYEI, and CYELMVI HBV 1.2mers (Figure 5a). After transfection with constructed and patient-derived clones into the Huh7 cells, HBV DNA replication was determined by Southern blot. Interestingly, as shown in Figure 5b, HBV DNA replication ability was lost in all clones containing the rtT301A mutation. The levels of secreted HBeAg were not significantly changed, whereas those of HBsAg varied among clones. Since HBV polymerase and surface genes overlap, mutations in the polymerase RT gene may cause corresponding mutations in the surface antigen open reading frame (ORF) (Table 2), altering surface antigenicity [12,13]. Therefore, loss of HBsAg level in Clone 4-7, which has two additional mutations in polymerase compared to Clone 1-29, may be attributed to the S34P or/and W199R mutations in the corresponding surface gene.

Similar results were obtained in HepG2 cells (Figure 5c). Quantitative real-time PCR also showed that considerable reduction in the replication ability of clones containing the rtT301A mutation commonly occurred in Huh7 and HepG2 cells (Figure 5d). The observed effect of rtT301A mutation on replication was comparable in both hepatoma cell lines. Concerning the characteristics of Huh7 and HepG2 cells that may contribute to their unique capacity to support viral replication [14,15], the effect of rtT301A mutation on HBV DNA replication was further evaluated in normal liver cell line (L-02). Unexpectedly, even though HBV surface antigens were secreted normally into culture supernatants (Figure 5e) viral replication could not be determined by Southern blot in L-02 cells, implying that essential host factors involved in supporting viral replication maybe lacking in L-02 cells. Of note, there seemed to be no significant changes in HBeAg and HBsAg levels between clones harboring WT and mutation in rtT301 position, except Clone 4-7 (Figure 5e).

Next, we examined whether the defective replication of the rtT301A mutant is due to impeded viral transcription. To examine the effect of rtT301A mutation on the level of HBV transcripts, Northern blot analysis was carried out in Huh7 cell line. As shown in Figure 5f, unlike attenuated replication level, clones carrying rtT301A mutation showed relatively higher levels of intracellular HBV RNA. This is probably caused by the accumulation of RNA transcripts due to the impaired RT activity of rtT301A containing mutants. Exceptional transcription and replication competence of the 4-7 clone requires further study.

To further evaluate the effect of rtT301A mutation on tenofovir resistance, IC_50_ as well as IC_70_ values of WT and mutant clones were determined by quantitative real-time PCR (Figure 5g) and summarized in Table 3. The impact of the rtT301A mutation in different HBV backbones on replication ability and drug resistance were compared by plotting the fold resistance (IC_50_ and IC_70_) vs. relative replication of each clone using real-time PCR data (Figure 5h). Collectively, regardless of mutant construct, the rtT301A mutation decreased replication capacity and increased tenofovir resistance.

### 2.5. The WT Polymerase (WT Pol) Partially Rescued the Replication Defect of the rtT301A Mutant

**Figure 6:**
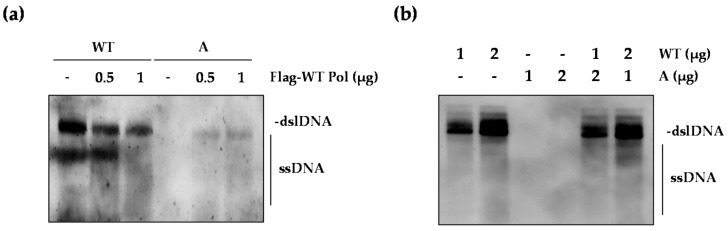
Impact of the HBV polymerase harboring rtT301A mutation on replication of WT HBV. (**a**) Effect of overexpressed WT Pol on the replication level of WT and rtT301A HBV mutant. Indicated plasmids were co-transfected into Huh7 cells. Total amount of plasmids was balanced with control vector. At three days post-transfection, cells were harvested for Southern blot. (**b**) Impact of rtT301A mutant on replication of WT HBV in Huh7 cells. Data in (**a**,**b**) were obtained from three independent experiments (mean ± SD).

Next, we asked whether WT polymerase (WT Pol) can rescue the replication-defective rtT301A mutant. To test this, the rtT301A mutant was co-transfected with increasing dose of the WT Pol construct (Figure 6a). Surprisingly, supplementation of WT Pol restored the replication of rtT301A mutant to some extent, whereas it reduced the replication of WT HBV. In addition, we examined the effect of the rtT301A mutant on the replication of WT HBV (Figure 6b). The replication of WT virus was not affected at all by the presence of rtT301A mutant virus, indicating that the rtT301A mutant Pol did not inhibit the function of WT Pol and may not act as a dominant-negative molecule. One possibility is that the rtT301A mutant pgRNA could be encapsidated along with WT Pol, therefore explaining the abundance of the rtT301A mutant in patient serum.

### 2.6. Impaired Replication Ability of rtT301A Mutants in Hepatoma Cell Lines Is Restored in PHHs

**Figure 7:**
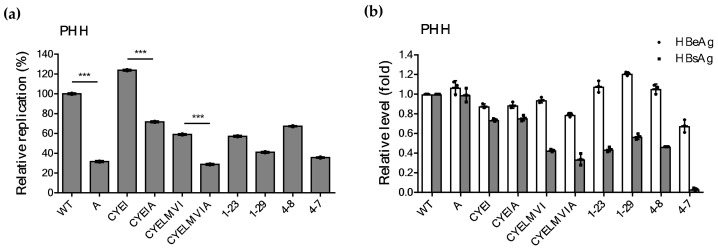
Effect of the rtT301A mutation on replication ability in primary human hepatocytes (PHHs). (**a**) The PHHs were transfected with 5 μg of indicated HBV replicons using Lipofectamine 3000. Four days later, the cells were harvested for the analysis. The replication capacity of each clone was determined by quantitative real-time PCR. *** *p* < 0.001. (**b**) Secreted HBeAg and HBsAg levels of indicated clones were determined by ELISA. Single-donor hepatocytes were used in this experiment. Data in (**a**,**b**) were obtained from three biological replicates.

Despite the impaired replication ability of rtT301A mutants harboring CYELMVI mutations, this mutant population was present in significant amount in CHB patients treated with TDF (Table 1). To explain this discrepancy between in vitro and in vivo results, the replicative ability of artificially constructed clones and patient-derived clones was evaluated in PHHs (Figure 6a). Surprisingly, clones carrying the rtT301A mutation significantly restored viral replication in PHHs (Figure 7a). They only reduced their replication ability by 40–50% as compared to their control clones. More importantly, regardless of the rtT301A mutation, there was no significant difference in replication levels among four patient-derived mutants (i.e., Clones 1-23, 1-29, 4-7, and 4-8). The levels of extracellular HBeAg and HBsAg were similar to that of the other cell lines (Figure 7b). The percentage of the replication competence of different mutant clones among the tested cell lines has been summarized in Table 4.

## 3. Discussion

TDF (or TAF) with a high genetic barrier to resistance is considered as the most potent antiviral agent for the treatment of chronic hepatitis B patients. Nevertheless, some recent studies have shown that rtS78T/sC69*, rtS106C/rtH126Y/rtD134E/rtL269I, and rtL180M/T184L/M204V/rtA200V are related to TDF resistance [11,16,17]. In this study, we presented a CHB patient case with suboptimal susceptibility to TDF treatment. Sequencing analysis revealed that the CYELMVI mutation, which we previously reported as a tenofovir-resistant mutation, was commonly observed in major viral populations. In addition, we found that the rtT301A substitution was concomitant with these mutations at a high rate. Thus, here we characterized the impact of this novel rtT301A substitution on replication ability and susceptibility to tenofovir treatment.

As TDF and TAF have the highest genetic barrier to resistance, a suboptimal virological response with or without virologic breakthrough is regarded as the first manifestation of antiviral drug resistance during treatment of CHB. A previous report of sequencing analysis of the HBV RT gene during the 8-year treatment of TDF revealed no tenofovir-resistant mutation and 70% of the virological breakthroughs were associated with non-adherence to medication [18]. There have also been reports of the efficacy and safety of TDF in lamivudine-resistant, ADV-experienced, and ETV-resistant patients with CHB [19,20,21,22]. Herein, we report a novel rtT301A mutation accompanying previously reported tenofovir-resistant mutations (CYELMVI) in a tenofovir-treated CHB patient without complete virological suppression.

During antiviral therapy, viruses with a mutation that has a replication advantage are selected and eventually become the predominant viral species, which clinically results in virological breakthrough. Viral fitness, which refers to the ability of a virus to replicate in a defined environment, is dependent on replication capacity and replication space [23]. The novel rtT301A mutation accompanying CYELMVI mutations resulted in increased resistance but a decrease in replication capacity. Discovery of the rtT301A mutation at the nadir point of HBV DNA during TDF treatment may have captured the evolution process of the tenofovir-resistant virus. A mutation-containing virus with low replication capability was present but unable to cause a virological breakthrough in this patient. On the contrary, as the patient stopped taking TDF for about 1 month, the WT virus with greater replication capacity was easily restored as the dominant population. After TDF was re-administered, the WT virus slowly declined in number and the virus with tenofovir-resistant mutation remained. Naturally occurring compensatory mutations in the HBV polymerase gene could reinstate the weakened replication capability caused by the drug-resistance mutations [24,25,26,27,28]. In ADV-treated patients, the reduced replication capacity of rtN236T mutant, which is well-known for ADV resistance, was restored by a compensatory mutation rtI233V [29]. Similar to the RT domain, the naturally occurring missense mutations in the TP domain that could result in impaired viral replication has occasionally been reported in the literature [30]. Possible compensatory mutations capable of restoring the disrupted replication by rtT301A is the next step in the evolutionary process of tenofovir-resistant mutations, which should be further explored.

An interesting clinical finding is that the tenofovir-resistant mutation was detected in this patient without virological or biochemical breakthrough. When the efficacy of TDF was defined as achieving HBV DNA <400 copies/mL at 240 weeks [18,19,20,21,22], 98.3% of patients with baseline HBV DNA of ≥9 log10 copies/mL and 99.2% of patients with baseline HBV DNA <9 log10 copies/mL achieved viral suppression in the pivotal trials [31]. Based on these reports, patients with partial virological response and without overt virological breakthrough were advised to adhere to the drug and wait for complete virological response to occur. However, henceforth, clinicians should suspect the possible development of tenofovir-resistant mutation in CHB patients without complete virological suppression for a prolonged period, despite good adherence to tenofovir-containing regimens and absence of the virological breakthrough.

HBV polymerase reportedly has a high mutation rate due to lack of the proofreading function. It is composed of four main domains—terminal protein (TP), spacer, RT, and RNase H—and their function and drug-resistant mutation in different HBV polymerase domains have been explored previously [32]. Drug-resistance-associated mutations of HBV polymerase often occur in the domains responsible for nucleotide recognition and strand synthesis, such as tyrosine–methionine–aspartate–aspartate (YMDD) [33]. However, the nucleotide position of rtT301A is far from the YMDD motif, indicating that this particular substitution might not directly affect binding and catalysis of polymerase substrates. Additionally, as its region is not overlapped with surface gene either, the effect of the rtT301A mutation may be solely attributed to itself. Nonetheless, the exact utility of the C-terminal RT domain, which is also known as the “thumb subdomain”, where the rtT301A mutation is located, has not been understood well. HBV polymerase carries out many functions related to the replication process, including viral RNA binding, RNA packaging, protein priming, template switching, DNA synthesis, and RNA degradation [32]. One study showed that the F501L substitution exhibited a decreased pregenomic RNA encapsidation level, resulting in defective DNA synthesis [34]. Thus, we believe that the rtT301A might be an important site in polymerase-mediated replication.

Another critical concept of this study is the consideration of the replication space. The drug susceptibility analysis in transient transfection showed that CYELMVI mutant clones from the patient were less susceptible to tenofovir as previously reported [11]. Quantitative real-time PCR exhibited abolished replication efficiency and enhanced tenofovir resistance in rtT301A-containing mutants in two hepatoma cell lines, Huh7 and HepG2. Nevertheless, predominant survival of HBV variants harboring the rtT301A CYELMVI mutation was present in the patient’s serum. In order to explain the discrepancy between in vitro study and clinical observation, similar experiments were performed in PHHs, which are the most physiologically relevant host cells. The abolished replication levels of clones with an rtT301A mutation from in vitro experiments in hepatoma cell lines were restored to a similar extent to the clones without rtT301A mutation in PHHs. This clinically relevant finding provides rationale that HBV variants bearing both CYELMVI and rtT301A could dominantly exist in the patient’s serum. One possibility is that the defective replication of rtT301A mutants might be compensated by unknown cellular factors in PHHs, which requires further examination. Considering that immortal hepatoma cell lines exhibited different replication competence, we assumed that intact cellular factor(s) in PHHs could be involved in supporting viral replication. In other words, the function of host protein(s), possibly interacting at the rtT301A mutation site, may be impaired in hepatoma cell lines, while they remained authentic in PHHs. There have been some reports unveiling the interaction between HBV polymerase and a number of host factors including Hsp90 [35], DDX3 [36], and APOBEC3G [37], some of which are incorporated into the nucleocapsid. Further investigation is required to know whether endogenous cofactors are possibly associated with HBV polymerase, and if so, whether their properties are altered by rtT301A substitution.

As the next step, molecular modeling of RT mutants would be helpful to elucidate the role and mechanism of the rtT301A mutation in viral fitness. Furthermore, identifying polymerase cofactors involved in the replication process, would shed light on new polymerase inhibitor targets for the development of novel antiviral drugs.

In summary, we herein show that the novel, naturally present rtT301A mutation significantly impaired viral replication, while slightly increasing tenofovir resistance in hepatoma cell lines. The reduced replication efficiency was restored in PHHs, which could explain why the replication-defective drug-resistant mutants determined in hepatoma cell lines are dominantly found in patients treated with antiviral agents. Although the incidence is quite low, we confirmed the presence of the CYELMVI mutation [11] in another CHB patient with clinical resistance to TDF. Overall, our data showed that the replication ability and drug susceptibility are different between hepatoma cell lines and physiologically relevant PHHs, probably due to a difference in the un-characterized essential host factors for viral fitness. Our findings may improve understanding of the mechanism involved in the viral replication process in HBV-infected patients.

## 4. Materials and Methods

### 4.1. Patient

This study is based on a patient who never achieved complete virological suppression during 4 years of TDF treatment at Chosun University Hospital, a university affiliated hospital, in Korea. Detailed flow of the patient is provided in Figure 1. A 47-year-old woman with CHB started ETV monotherapy (0.5 mg/day) in 2010. During the following 5 years of ETV monotherapy, the patient never achieved complete virological suppression (i.e., serum HBV DNA below 20 IU/mL). In January 2015, the antiviral treatment was changed to TDF (300 mg/day). At that time, the patient did not present mutation at any known sites for lamivudine, ADV, or ETV. After 4 years of TDF treatment, the patient still did not achieve complete virological suppression, and the nadir for serum HBV DNA level was 54,395 IU/mL. The HBV viral load fluctuated between 5.4 × 10^4^ and 1.2 × 10^9^ IU/mL. Drug compliance was assessed by inquiry to the patient at each visit for taking the medication and the medication possession ratio. The report of a novel, quadruple mutation associated with tenofovir resistance led to the examination for the presence of mutation in this patient [11]. The patient provided written informed consent before enrollment in the study, which was approved by the Institutional Review Board of Chosun University Hospital (IRB no. 2020-05-004, approval date: 22 May 2020).

### 4.2. HBV RT Sequence Analysis

To analyze the HBV variants during TDF treatment, blood samples were collected four times, and viral DNA was extracted from the patient’s serum using QIAmp MinElute Virus Spin Kit (QIAGEN) according to the manufacturer’s instructions. To specifically amplify the RT region, we performed PCR (FastStart High fidelity PCR system, Roche, Mannheim, Germany) using viral DNA as a template, with primers spanning the entire RT gene [11]. Primers and conditions for amplification were as follows; forward 5′-AAT CTC GAG GAC TGG GGA CCC TGC ACC-3′ (XhoI site is underlined); reverse 5′-GAG CAG CCA TGG GAA GGA GGT GTA TTT CCG -3’ (NcoI site is underlined), 95 °C for 5 min followed by 32 cycles of 95 °C for 40 s, 60 °C for 50 s, and 72 °C for 10 min. The purified PCR products were ligated into the pGEM-T vector (pGEM-T Vector Systems, Promega, Madison, WI, USA), and more than 10 clones were isolated and sequenced. Mutations in the RT domain were analyzed as compared to a reference HBV sequence (wild-type (WT) genotype C, National Center for Biotechnology Information (NCBI) accession number: GQ872210).

### 4.3. Construction of HBV Reverse Transcriptase (RT) Mutant Replicons Harboring Artificially Substituted or Patient-Derived RT Domains by Site-Directed Mutagenesis 

Two clones (CYEI: rtS106C (C) + rtH126Y (Y) + rtD134E (E) + rtL269I (I) and CYELMVI: rtS106C (C) + rtH126Y (Y) + rtD134E (E) + rtV173L (L) + rtL180M (M) + rt204V (V) + rtL269I (I)) have been described in our previous study [11]. The RT gene was amplified by PCR and cloned into the HBV 1.2mer replicon and sequenced. The rtT301A substitution was introduced to the WT, CYEI, and CYELMVI replicons by NEBuilder HiFi DNA assembly Cloning Kit (New England Biolabs, Ipswich, England) according to the manufacturer’s protocol. For site-directed mutagenesis, the vector fragment was amplified by PCR using the following primers: forward 5′-GCT GCT AGG CTG TGC TGC CAA C-3′, reverse 5′-GAA GAT TGA CGA TAA GGG AGA GGC AGT AG-3′. Two insert fragments were also prepared and cloned simultaneously to produce HBV 1.2mer replicon using the following primers: Fragment 1 (forward 5′-CTC CCT TAT CGT CAA TCT TCT CGA GGA CTG GGG ACC CTG-3′, reverse 5′-CAA AGC AGG ATA GCC ACA TTG TGC AAA AGG GGC-3′, Fragment 2 (forward 5′-GCC CCT TTT GCA (T to A amino acid change) CAA TGT GGC TAT CCT GCT TTG-3′, reverse 5′-TGG CAG CAC AGC CTA GCA GCC ATG GGA AGG AGG TGT ATT TCC G-3′). The patient-derived RT products were amplified from viral DNA using forward fragment 1 and reverse fragment 2 primers and were cloned into the HBV 1.2mer replicon by NEBuilder HiFi DNA assembly Cloning Kit (New England Biolabs). All generated clones were confirmed by sequencing.

### 4.4. Isolation of Primary Human Hepatocytes (PHHs)

A virus-free human liver tissue specimen was obtained from therapeutic hepatectomy. Informed consent was obtained from a patient (66-year-old male) prior to surgery, which was approved by the Institutional Review Boards at Korea University Hospital (IRB no. ED10287). PHHs were isolated by a two-step collagenase perfusion method as described previously [38]. Briefly, the liver specimen was perfused through small vessels on the cut surface of the specimen with perfusion buffer supplemented with collagenase (0.5 g/L) and calcium chloride (0.56 g/L). The liver was filtered through stainless steel meshes (grid size 500, 300, 150 μm), and the cell pellets were washed twice with Dulbecco’s Modified Eagle’s medium (DMEM; Welgene, Gyeongsan, South Korea) supplemented with 10% fetal bovine serum (FBS; Capricorn, Ebsdorfergrund, Germany) and 1% penicillin/streptomycin (Gibco, Grand Island, NY, USA) at 50*g* for 5 min. Isolated PHHs were resuspended in William’s E medium (Gibco) and plated on six-well collagen-coated plates (Corning, Tewksbury, MA, USA) for HBV infection.

### 4.5. Cell Culture, Transfection, and Drug Treatment

Two human hepatoma cell lines (Huh7 and HepG2) and a normal hepatocyte cell line (L-02) were obtained from American Type Culture Collection (ATCC) and cultured in DMEM (Welgene) supplemented with 10% FBS (Capricorn) and 1% penicillin/streptomycin (Gibco) at 37 °C in a 5% CO_2_ incubator. Approximately 8 × 10^5^ cells were seeded into six-well plates. On the following day, the cells were transfected with 2 μg of HBV 1.2mer clones using Lipofectamine 2000 reagent (Invitrogen, Carlsbad, CA, USA). After 5 h, tenofovir (kindly provided by Dong-A Pharmaceutical Co., Korea) was administered daily at the indicated concentrations in fresh medium. At four days post-transfection, cells were harvested for Southern blot analysis, and supernatants were collected and analyzed for the secretion of HBV antigens (HBeAg and HBsAg) by ELISA. The Flag-tagged WT HBV polymerase was described in our previous study [39].

PHHs were maintained in William’s medium E (Gibco) containing cell maintenance supplements (CM4000; Gibco), 2% FBS, and 1% penicillin/streptomycin (Gibco). Approximately 3 × 10^6^ cells were seeded into collagen-coated six-well plates (Corning). On the next day, the cells were transfected with 5 μg of HBV 1.2mer clones using Lipofectamine 3000 (Invitrogen) according to the manufacturer’s instruction. Four days after transfection, the cells were harvested for analysis.

### 4.6. Southern and Northern Blot Analysis

HBV replication was analyzed by Southern blot as described previously with some modifications [11,13]. Briefly, cells were lysed with HEPES buffer (10 mM HEPES, 1mM EDTA, 100 mM NaCl, and 1% NP-40), and after centrifugation, lysate was treated with DNase I (Roche, Mannheim, Germany) to digest the transfected plasmid DNA. Then the intracellular HBV capsids were precipitated overnight with 7.4% polyethylene glycol solution (PEG 8000; Sigma). HBV capsid breakdown and DNA release was obtained by proteinase K (Roche) in the presence of 0.5% sodium dodecyl sulfate (SDS), followed by phenol/chloroform/isoamyl alcohol (25:24:1) extraction and ethanol/sodium acetate precipitation. Three-quarters of the DNA was separated on 1% agarose gel and transferred onto a positively charged Hybond-XL membrane (GE Healthcare, Buckinghamshire, UK) by alkaline transfer method. To detect HBV replicates, we synthesized seven fragments of digoxigenin (DIG)-incorporated HBV probe using PCR DIG Probe Synthesis Kit (Roche), which were designed to target the HBV whole genome with 200–300 bp length, respectively [40]. The membrane was hybridized with DIG-probe in Church buffer containing 1% BSA, 7% SDS, 0.5M Na_2_HPO_4_ (pH 7.2), and 1 mM EDTA. HBV DNA was detected using DIG Nucleic Acid Detection Kit (Roche) according to the manufacturer’s protocol. The signals were detected by ImageQuant 800 (Amersham, Buckinghamshire, UK).

Northern blot was performed to determine the levels of HBV transcripts. Briefly, 20 μg of total RNA, which was extracted by TRIzol reagent (Invitrogen), was electrophoresed on 1% formaldehyde-agarose gel, and transferred to a positively charged Hybond-XL membrane. After UV crosslinking, membrane was hybridized with the same probe used for Southern blot. RNA signal was similarly detected using a DIG Nucleic Acid Detection Kit (Roche).

### 4.7. Quantitative Real-Time PCR

A quarter of the total DNA extracted from intracellular HBV particles was used for quantification. For quantification of HBV RNA transcription, 2 μg of total RNA was synthesized into cDNA using a High-Capacity RNA-to-cDNA Kit (Applied Biosystems, Foster City, CA, USA) according to the manufacturer’s instructions. Each reaction mixture (15 μL) contained 2 μL of DNA (10- or 20-fold diluted) or cDNA (4-fold diluted), 0.4 μM of each primer, and 7.5 μL of SYBR green master mix (Applied Biosystem). Amplification conditions were 95 °C for 10 min, followed by 40 cycles of 95 °C for 15 s, and 60 °C for 1 min in QuantStudio 3 Real-Time PCR System (Applied Biosystem). Relative replication levels were calculated by the comparative 2^−∆∆CT^ method [11,13]. The levels of HBV RNA were normalized to *GAPDH* gene (NCBI reference sequence: NC_000012.12). PCR primers were as follows: HBV DNA (or RNA); forward 5′-CTC GTG GTG GAC TTC TCT C-3′, reverse 5′-CTG CAG GAT GAA GAG GAA-3′ GAPDH; forward 5′-ATC ATC CCT GCC TCT ACT GG -3′, reverse 5′-TGG GTG TCG CTG TTG AAG TC-3′.

### 4.8. ELISA

To measure the levels of secreted HBV antigens (HBeAg and HBsAg), culture supernatants were collected before harvesting the cells. The supernatants were diluted (Huh7: 20- and 100-fold; HepG2: 5- and 10-fold; PHH: undiluted) for HBeAg and HBsAg, respectively) and subjected to an enzyme-linked immunosorbent assay (ELISA) using a kit (Wantai Pharm Inc., Beijing, China) following manufacturer’s instructions. The optical density was measured at 450 nm using a spectrophotometer (SpectraMax Plus 384).

### 4.9. Statistical Analysis

At least three independent experiments were performed for analysis. Data are mean ± SD. Statistical significance was evaluated by Student’s *t*-test in GraphPad Prism v6: * *p* < 0.05; ** *p* < 0.01; *** *p* < 0.001.

## Figures and Tables

**Table 1:** Identification of HBV RT mutation isolated from a TDF-treated patient.

Blood Sample		Amino Acid at RT Position																																																
	HBV Clone	8	12	38	42	55	106	110	117	126	128	134	148	164	167	173	180	181	190	191	199	204	207	214	223	226	226	238	241	247	266	267	269	270	274	275	292	293	295	300	301	313	317	320	323	324	329	330	332	336
	Wild-Type (GQ872210)	E	H	T	L	H	S	R	S	H	T	D	Y	L	R	V	L	A	V	V	L	M	V	V	S	N	N	N	K	L	V	Q	L	K	R	K	G	L	G	F	T	A	S	A	F	S	A	F	C	L
**#1**	1-1																																														G			
	1-4				P		C			Y		E			L	L	M					V	A									L	I								A									
	1-5			A															I					A			H				I												A							
	1-6,7,8																M					V																												
	1-9													P																		L											A							M
	1-10																																					P												
	1-17						C			Y		E				L	M					V											I		Q	R				C										
	1-19						C			Y		E			H	L	M					V											I								A								W	
	1-23,28						C			Y		E				L	M					V											I																	
	1-29,30,31,38						C			Y		E				L	M					V										L	I								A							S		
	1-32																																																	
	1-33																								P																									
	1-34						C			Y		E				L	M					V										L	I								A								G	
	1-35,37			A		R														I							H				I												A							
	1-36		R	A																							H				I	L											A							
**#2**	2-1			A		R														I						H					I	L											A				T			
	2-2			A			C				N															H					I	L											A							
	2-3			A												L					V					H					I	L											A		L					
	2-4			A		R																				H					I	L											A							
	2-6			A			C																			H					I	L											A							
	2-7			A																I						H				P	I	L											A				T			
	2-8			A			C													I						H					I	L											A				T			
	2-9			A			C																			H					I	L											A							
	2-11			A		R	C																			H					I	L											A							
	2-12			A					F											I						H					I	L											A							
**#3**	3-1			A																						H					I	L										A								
	3-2			A																						H					I	L										A		T						
	3-3			A																						H					I	L										A					T			
	3-4			A		R	C	G					F													H					I	L										A								
	3-5			A																I						H					I	L										A					T			
	3-7			A		R																				H					I	L					S					A								
	3-9			A														T								H					I	L										A								
	3-10			A		R																				H					I	L										A								
**#4**	4-1																																																	
	4-2			A			C																						N			L											A							
	4-3																																	E					S											
	4-4	K																				V																								P				
	4-7				P		C			Y		E				L	M					V	A									L	I								A									
	4-8						C			Y		E				L	M					V											I																	
	4-11			A			C																			H					I	L											A							
	4-12													P						I								S																						
	4-14			A																						H					I	L															T			

Columns with gray background highlight representative tenofovir-resistant mutations (CYELMVI) and rtT301A mutation.

**Table 2:** Commonly found HBV RT mutations and corresponding surface mutations in patient-derived viral mutants.

Clone	Region	Mutations in the Corresponding Gene											
**WT**	**RT**	**L42**	**S106**	**H126**	**D134**	**V173**	**L180**	**M204**	**V207**	**Q267**	**L269**	**T301**	**F330**
	**S**	**S34**	**L98**	**T118**	**I126**	**W165**	**W172**	**W196**	**W199**	**-**	**-**	**-**	**-**
**1-23**	**RT**		C	Y	E	L	M	V			I		
	**S**		V	-	S	-	-	-					
**1-29**	**RT**		C	Y	E	L	M	V		L	I	A	S
	**S**		V	-	S	-	-	-					
**4-8**	**RT**		C	Y	E	L	M	V			I		
	**S**		V	-	S	-	-	-					
**4-7**	**RT**	P	C	Y	E	L	M	V	A		I	A	
	**S**	P	V	-	S	-	-	-	R				

**Table 3:** Determination of IC_50_ and IC_70_ values of mutant clones against tenofovir in Huh7 cells.

Clone	Huh7			
	IC_50_	IC_70_	Fold Resistance (/IC_50_)	Fold Resistance (/IC_70_)
WT	4.20 ± 0.24	8.05 ± 0.45	1	1.00
A	12.10 ± 3.48	31.00 ± 1.25	2.88	3.85
CYEI	36.20 ± 5.93	53.50 ± 0.85	8.62	6.65
CYEIA	41.00 ± 3.13	81.50 ± 0.56	9.76	10.12
CYELMVI	41.20 ± 2.03	81.90 ± 1.30	9.81	10.17
CYELMVIA	46.80 ± 2.21	88.50 ± 0.68	11.14	10.99
1-23	43.60 ± 4.30	>50	10.38	>11.90
1-29	44.15 ± 2.25	>50	10.51	>11.90
4-8	41.26 ± 1.96	>50	9.82	>11.90
4-7	>50	>50	>11.90	>11.90

The quantitative real-time PCR experiment was repeated three times as independent biological replicates.

**Table 4:** Relative replication ability of mutant clones among Huh7, HepG2 cells, and PHHs compared to that of wild type.

Clone	Replication Ability (%)		
	Huh7	HepG2	PHH
WT	100	100	100 ± 0.93
A	4.51 ± 0.73	16.45 ± 1.96	31.6 ± 0.97
CYEI	116.18 ± 2.61	125.09 ± 2.14	123.84 ± 0.90
CYEIA	4.81 ± 0.71	25.69 ± 9.63	71.7 ± 0.93
CYELMVI	75.50 ± 0.50	85.26 ± 5.26	58.93 ± 0.85
CYELMVIA	8.26 ± 2.06	23.79 ± 1.79	28.76 ± 0.96
1-23	79.00 ± 1.00	81.52 ± 1.02	57.12 ± 0.58
1-29	5.50 ± 1.50	25.62 ± 3.00	40.96 ± 0.72
4-8	81.13 ± 2.13	84.49 ± 0.75	67.26 ± 0.88
4-7	2.50 ± 0.50	15.90 ± 2.77	35.63 ± 0.96

Values were determined by three independent quantitative real-time PCR experiments.

## Data Availability

The data presented in this study are available on request from the corresponding author. The data are not publicly available due to privacy of research participants.
